# Development of Impurity-Detection System for Tracked Rice Combine Harvester Based on DEM and Mask R-CNN

**DOI:** 10.3390/s22239550

**Published:** 2022-12-06

**Authors:** Zhuohuai Guan, Haitong Li, Xu Chen, Senlin Mu, Tao Jiang, Min Zhang, Chongyou Wu

**Affiliations:** Nanjing Institute of Agricultural Mechanization, Ministry of Agriculture and Rural Affairs, Nanjing 210014, China

**Keywords:** impurity rate, combine harvester, rice, discrete element method, Mask R-CNN

## Abstract

Impurity rate is one of the key performance indicators of the rice combine harvester and is also the main basis for parameter regulation. At present, the tracked rice combine harvester impurity rates cannot be monitored in real time. Due to the lack of parameter regulation basis, the harvest working parameters are set according to the operator’s experience and not adjusted during the operation, which leads to the harvest quality fluctuating greatly in a complex environment. In this paper, an impurity-detection system, including a grain-sampling device and machine vision system, was developed. Sampling device structure and impurity extraction algorithm were studied to enhance the impurity identification accuracy. To reduce the effect of impurity occlusion on visual recognition, an infusion-type sampling device was designed. The sampling device light source form was determined based on the brightness histogram analysis of a captured image under different light irradiations. The effect of sampling device structures on impurity visualization, grain distribution, and mass flow rate was investigated by the discrete element method (DEM). The impurity recognition algorithm was proposed based on Mask R-CNN, which mainly includes an impurity feature extraction network, an ROI generation network, and a target segmentation network. The test set experiment showed that the precision rate, recall rate, average precision, and comprehensive evaluation indicator of the impurity recognition model were 92.49%, 88.63%, 81.47%, and 90.52%, respectively. The conversion between impurity pixel number and its actual mass was realized according to the pixel density calibration test and impurity rate correction factor. The bench test result showed that the designed system has a good detection accuracy of 91.15~97.26% for the five varieties. The result relative error was in a range of 5.71~11.72% between the impurity-detection system and manual method in field conditions. The impurity-detection system could be applied to tracked rice combine harvesters to provide a reference for the adjustment of operating parameters.

## 1. Introduction

The combine harvester is the most widely used agricultural equipment to efficiently accomplish harvesting work, such as rice, wheat, etc. The harvested grain inevitably contains unwanted impurities, including straw, chaff, and light miscellaneous. The impurities in harvested grain directly affect the workload and production costs of subsequent processing. Impurity rate is the ratio of material mass other than grain to grain mass, which is one of the key performance indicators of the combine harvester and is also the key basis for regulating the threshing and cleaning system [1,2,3]. The detection of the impurity rate during harvesting in real time is conducive to work parameter adjustment and improving the harvesting quality [4,5]. However, the tracked rice combine harvester impurity rates cannot be monitored in real time at present. Due to the lack of parameter regulation basis, the harvest working parameters are set according to the operator’s experience and not adjusted during the operation [6,7]. The harvest quality fluctuates greatly in a complex environment due to the lack of timely information on the impurity rate. The development of a combine harvester impurity-detection system is of great significance to improve harvesting performance. The improvement in impurity detection and other intelligent technology will improve agricultural performance [8].

Technologies, such as machine vision and spectral detection, have been applied to distinguish and quantify the rice and impurity in the grain [9]. The spectral detection method can overcome the effect of material masking, but its adaptability to the random state of the material is weak, such as material moisture content and maturity [10,11]. Yu et al. used a convolutional neural network to process hyperspectral images to achieve a nondestructive detection of imperfect grains and the overall recognition rate of the test set reached 99.98% [12]. Chen et al. preferred the characteristic wavelengths of wheat sample spectra with different indicators by principal component analysis and contribution weight coefficients, and a least squares support vector machine was applied to construct inverse models of the impurity rate of wheat samples based on different indicators. The experimental results showed that the modeling coefficient of determination of the inverse model of impurity rate was greater than 0.9, the validation coefficient of determination was greater than 0.85, the root mean square error was less than 0.29, and the relative analysis error was greater than 2 [13].

The machine vision method is well adapted to the material and is the main method to visualize the quality of the grain. Wallays et al. developed a machine-vision-based wheat impurity rate detection sensor that can monitor the impurity rate of harvested wheat online and display the monitored value on an interface [14]. Mahirah et al. designed a dual-light-source impurity detection device and its accompanying image processing algorithm for detecting impurities and broken kernels in grains with a measurement accuracy of >75% [15,16]. Pourreza et al. investigated the distinction between wheat seeds and impurities in texture features and used a linear judgment analysis classifier to classify the features with 98.15% accuracy in impurity differentiation [17]. Kayabasi et al. produced a wheat seed dataset using ANN neural network and Bayesian regularization learning algorithm from the length, width, area, and perimeter to provide a machine vision system equipped with dual illumination and image-processing algorithms for detecting and measuring undesirable objects and damaged grains in harvested rice grains [18]. Chen et al. optimized the irradiation and installation method of the LED light source to obtain high-quality images by image histogram, identified the grain and impurity morphologically, and measured the thousand-grain mass, stalk surface density, and fine stalk line density of rice at the same time, and established a mathematical model to calculate the impurity mass of grain. The visualized tree was used to classify the particles labeled in the binary image, so the impurity in a range of 0 to 2.88% can be monitored [19,20,21]. Momin et al. used the HSI color model to segment the image background and, subsequently, of soybean, dockage fractions were detected using median blurring, morphological operators, watershed transformation, and component labeling based on projected area and circularity; the identification accuracy of dockage fractions was 96% [22].

In view of the above research, grain sampling and machine vision were mainly methods used for real-time impurity rate detection in a combine harvester. However, machine vision can only identify the impurity on the surface of the grain, and the mutual occlusion between the rice and impurity has a great influence on the detection results. The extraction of impurity by color space is also prone to misidentification when it is close to the rice. Therefore, an infusion-type sampling device was developed to reduce the effect of impurity occlusion on visual recognition, and impurity extraction algorithms based on Mask-R-CNN were developed to enhance the impurity identification accuracy. Bench test and field test were conducted to evaluate the impurity-detection system.

## 2. Materials and Methods

### 2.1. Impurity-Detection Method for Rice Combine Harvester

**Figure 1:**
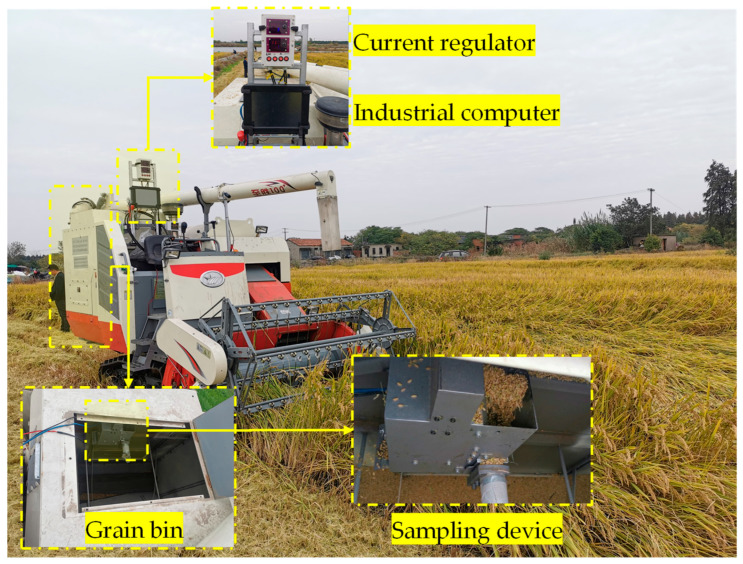
Impurity-detection system for tracked rice combine harvester.

**Figure 2:**
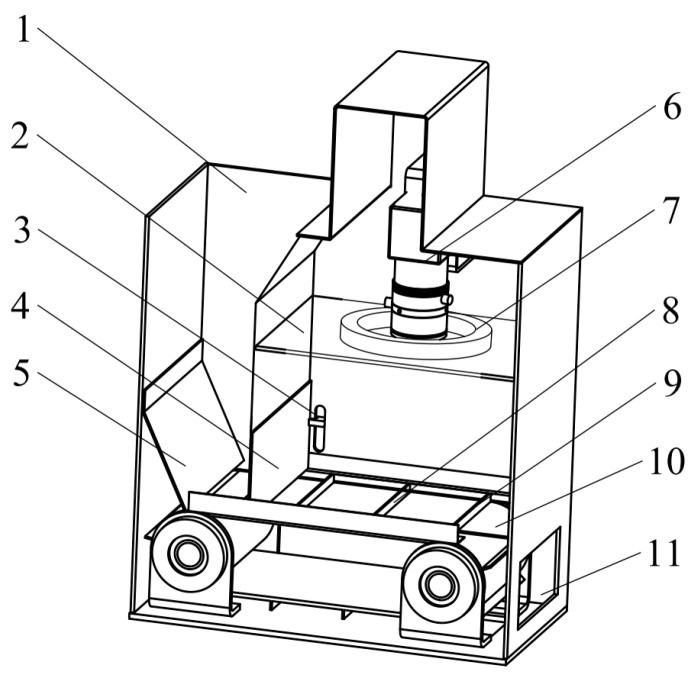
Structure diagram of infusion-type sampling device. (1) Inlet, (2) dust baffle, (3) flow-rate-adjustment lever, (4) deflector, (5) baffle, (6) camera, (7) light source, (8) conveyor belt, (9) corrugation, (10) transparent platen, and (11) outlet.

The impurity-detection system mainly includes infusion-type sampling device, current regulator, industrial computer, and related processing software, as shown in Figure 1. Harvested grain sampling, grain image analysis, and impurity rate calculation function can be implemented. The sampling device (Figure 2) was installed in the grain bin, under the grain auger. Some of the grain discharged by the auger falls into the sampling device inlet and then transported by conveyor belt to the outlet and, finally, discharged back into the grain bin. During this process, the camera captures the grain on the conveyor belt to achieve continuous image acquisition. The rice and impurity in the image are identified based on Mask R-CNN. The amount of rice and impurity in pixels is obtained via pixel statistics. The actual mass of rice and impurity is estimated based on material pixel density calibration test and the actual impurity rate can be calculated.

### 2.2. Structure Analysis of Infusion-Type Sampling Device

Sampling is an important part of the impurity rate detection. To minimize the mutual shading between impurity and rice, an infusion-type sampling device was designed, as shown in Figure 2 [error]. The sampling device is made of steel and has a length, width, and height of 210 mm, 295 mm, and 110 mm, respectively, which mainly includes a deflector, conveyor belt, camera, light source, etc. The conveyor belt is driven by the DC motor with a speed sleeplessly adjustable (0~0.25 m/s). The LED light source brightness and conveyor belt speed were adjusted by the current regulator. The gap between the deflector and the conveyor belt can be adjusted in a range of 0~30 mm. The deflector plate can limit the material flow and a thin layer of around one kernel thick could be created, so that the mutual occlusion of impurity and rice in the material flow is reduced, which would improve the detection accuracy of impurity rate. The dust baffle is made of plexiglass to avoid dust in the grain covering the lens. The conveyor belt is equipped with corrugation to enhance the grain-conveying capacity and prevent accumulation at the deflector. The corrugation has a height and interval of 5 mm and 50 mm. Under the conjunction of conveyor belt, corrugation, and transparent platen, the material is completely constrained in the set space and can be effectively sampled in face of the working vibration of the harvester.

Daheng MER-132-43U3C CCD camera and VS-LDA4 zoomable lens were used in combination to capture grain images. The vertical distance between the lens and the conveyor belt is 120 mm. The resolution of the camera was 1292 pixels × 964 pixels. The impurity rate detection program was installed on an industrial computer. The collected grain image and impurity identification result could be displayed and saved in real time. The industrial computer processor, graphics card, and system memory were Intel Core i7-4790S, Intel HD Graphics 4600, and 8 GB, respectively.

#### 2.2.1. Light Irradiation

**Figure 3:**
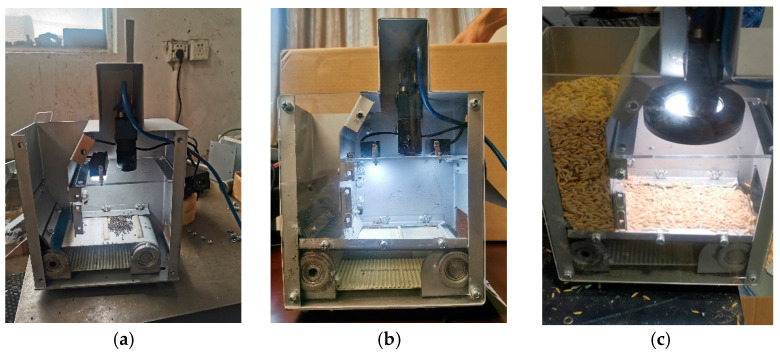
Different light irradiations of sampling device: (**a**) single-sided-strip LED, (**b**) double-sided-strip LED, (**c**) central-ring LED.

The light irradiation affects the color space of the grain image and the subsequent impurity recognition algorithm. To investigate the effect of light irradiation on the grain image, single-sided-strip LED (with a power of 4.5 W and max spectrum of 70,000 LUX), double-sided-strip LED (each strip LED with a power of 4.5 W and max spectrum of 35,000 LUX) and central-ring LED (with a power of 4.5 W and max spectrum of 70,000 LUX) were tested as shown in Figure 3. The HSV (Hue Saturation Value) color space model was used to analyze the light uniformity of grain images captured under different light irradiations. The *V* component in the HSV model is the normalized value of the lightness, ranging from 0 to 1, and it is uncorrelated with light intensity. The effect of light intensity on brightness and uniformity can be excluded. By comparative analysis of the image *V* value distribution histogram and its coefficient of variation, the optimal light source could be determined [23].

#### 2.2.2. Light Irradiation

The conveyor belt speed correlates with the sample frequency and the image quality. If the conveyor belt speed is too slow, the sampling interval is large and the total sample size is small. However, there will be trailing shadows in the image if the conveyor belt speed is too fast, affecting the recognition of impurity. To avoid motion blur, the distance of object movement within the exposure time is generally required to be less than one pixel in the image. The conveyor belt speed should meet the condition:

*vt* < *H*/*h* (1)

where *v* is the conveyor belt speed, m/s; *t* is the exposure time, 0.7 ms in this paper; *H* is the width of visual field in the direction of belt movement, 0.148 m in this paper; *h* is the image pixel number in the direction of belt movement, 1292.

According to Equation (1), the conveyor belt speed should be less than 0.164 m/s. The conveying speed was determined to be 0.15 m/s to balance the material flow rate and image quality.

#### 2.2.3. The Gap between the Deflector and the Conveyor Belt

The deflector was developed to limit the grain flow and the thickness of grain accumulation on the conveyor belt. The gap between the deflector and the conveyor belt affects the thickness of the material on the conveyor belt, impurity obscuration situation, and the grain mass flow rate. If the gap is too narrow, the material flow will be hindered, but cannot achieve the function of reducing impurity shading when the gap is too large. DEM simulation was used to investigate the effect of the gap on grain transport in this paper. The accumulation and obscuration of the grain can be directly observed by simulation. After simulation, the grain image was intercepted and binarized. The occlusion of impurity was quantified by the occlusion rate. The calculation equation was:

(2) $$S=\frac{N_{pt}-N_{pr}}{N_{pt}}\times 100\%$$

where *S* is the impurity occlusion rate; *N_pt_* is number of impurity pixels without occlusion; *N_pr_* is actual impurity pixel number.

### 2.3. Grain Transport Analysis Based on DEM

#### 2.3.1. DEM Model

**Figure 4:**
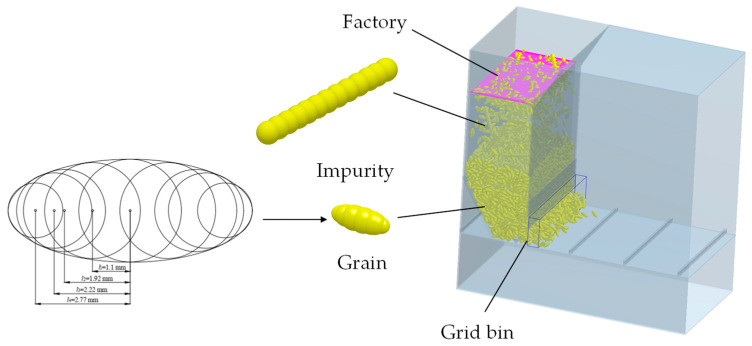
EDEM simulation model.

The distribution behavior of the grain in the sampling device was further studied by DEM. The 3D computer-aided design (CAD) models of the sampling device were imported into the DEM software EDEM 2018 (DEM Solutions Limited, Edinburgh, UK). The 3D simulation models and boundary conditions were determined by geometry parameters and working parameters, respectively. The material other than grain (MOG) in the grain bin mainly includes cylindrical short stem and fine light residual. The mutual occlusion between short stem and rice was mainly investigated because the occlusion between the fine light residual and rice can be neglected. A 9-sphere filling model was constructed to simulate rice in this paper. The outer envelope of the 9 spheres was ellipsoidal with a long axis of the ellipse 7.20 mm and a short axis 3 mm. The sphere was symmetrical from left to right, the distance from the center of the left 4 spheres to the center of the ellipsoid was 1.10 mm, 1.92 mm, 2.2 mm, and 2.77 mm, respectively, and the spheres’ radius from left to right was 0.80 mm, 1.10 mm, 1.20 mm, 1.40 mm, 1.50 mm, 1.40 mm, 1.20 mm, 1.10 mm, and 0.80 mm, respectively [24]. The short stalk model is filled with multiple spheres with a cylindrical outer envelope of 35.00 mm in length and 3.40 mm in diameter [25]. The simulation model is shown in Figure 4.

The density, Poison’s ratio, and shear modulus of rice, impurity, belt, and shell are shown in Table 1 [26,27]. According to the general impurity rate of the rice combine harvester, the mass ratio of the impurity to rice was set as 0.03:1. The particle factory was set up for 2 kg of particles generated statically and the simulation time was 10 s.

The contact relations in the simulation include six types, rice–rice, rice–impurity, rice–belt, rice–shell, impurity–belt, and impurity–shell. The contact parameters between the materials are detailed in Table 2 [28,29]. The Hertz-Mindlin (no slip) model was chosen as the particle contact model in the EDEM software for the materials. The simulation time step, environmental gravitational acceleration, and preservation interval were 0.5 × 10^−6^ s, 9.8 m/s^2^, and 0.01 s, respectively.

#### 2.3.2. Simulation Design and Validation

Simulation tests were conducted with the deflector gap as the variable. The grain flow rate and the occlusion rate were taken as the evaluation indicators. The grain flow rate could be counted by the software post-processing function and the occlusion rate was calculated according to Equation (2). Based on the geometry of rice and impurity, the test range of the deflector gap was determined to be 7.5 mm~20.0 mm with a variation of 2.5 mm. To reduce the influence of random factors such as material generation location and morphology on the result, the tests were repeated three times at each parameter level and averaged.

To verify the simulation result, a physical test of grain flow rate was carried out at the same parameters. The actual grain mass flow rate was measured by placing a sample box at the outlet of the sampling device. The grain discharge from the sampling device was collected and weighed within 10 s after the grain flow was stabilized. The actual grain mass flow rate was calculated and compared with the simulation result.

### 2.4. Impurity Recognition Algorithm Based on Mask R-CNN

#### 2.4.1. Overall Methodology

**Figure 5:**
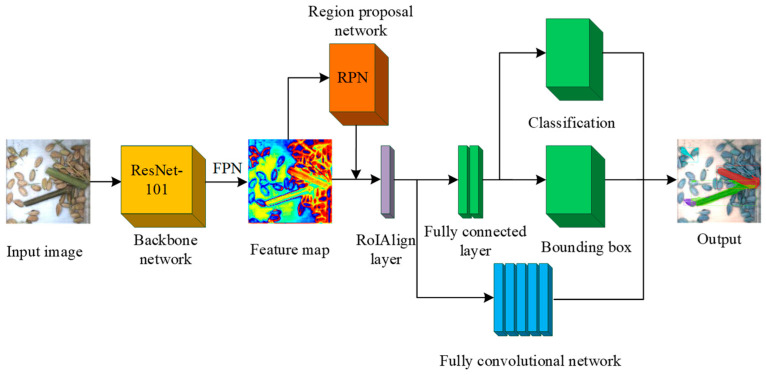
Flowchart of rice impurity recognition algorithm based on Mask R-CNN.

The Mask R-CNN method is based on the Faster R-CNN structure by adding a mask branch that predicts the segmentation target mask, in parallel with the classification and regression branches, so that the Mask R-CNN has the function of instance segmentation [27]. The Rol Pooling layer is replaced by the Region of Interest Align layer (RoIAlign). The coordinates of the feature image and the input pixels are aligned to achieve pixel-level segmentation. In this paper, the grain image feature was recognized by the ResNet-101 network and the corresponding feature map was obtained. Anchor boxes were generated by Region Proposal Network (RPN) and pooled into fixed-size feature maps with RoIAlign. The Mask segmentation of rice impurity was completed by the classification branch (CLS) inside the RPN. Finally, the impurity prediction results were output and the rice pixel number and impurity pixel number were obtained. The developed impurity recognition algorithm flowchart is shown in Figure 5.

#### 2.4.2. Image Annotation and Dataset Production

The designed infusion-type sampling device was installed in the combine harvester to capture grain images during harvesting and the images were used to investigate the impurity recognition algorithm. A total of 1200 grain images was captured during rice harvesting by the designed sampling device, from which 500 images were randomly selected for labeling as the training set and the remaining images were used for the testing set.

**Figure 6:**
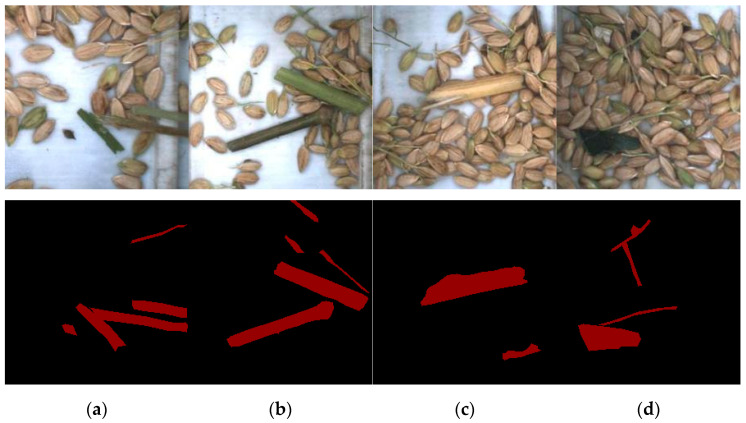
The captured grain image and the impurity mask image. (**a**) Fine impurities; (**b**) coarse impurities; (**c**) impurities approximating the color of rice; (**d**) irregular shape impurities.

The images were cropped and scaled to 224 pixels × 224 pixels, and the edge contours of the rice and impurity were manually marked using the image labeling tool LABELME [28]. The annotation labels and coordinates were saved to the corresponding JSON files. The label image was divided into 2 parts: the area inside the tag is the target and other areas are considered as background. To increase the sample size of the training set and strengthen the stability of the model, the training set was expanded to 1500 by changing the brightness and mirroring. The grain image and the impurity mask image are shown in Figure 6.

#### 2.4.3. Impurity Feature Extraction Network

Convolutional neural network model with different weight layers can be designed in the Mask R-CNN. The network accuracy increases with the number of layers of the network, but the training time also increases. The residual network does not increase the model parameters, which can solve the training degradation problem and improve the model convergence. ResNet-101 deep residual network with 5 network module layers combined with FPN was used as the backbone feature extraction network in this paper. The network structure is shown in Table 3.

#### 2.4.4. Generation of RoIs and RoIAlign

A fixed-size sliding window was used to scan obtained impurity feature image. An n-dimensional length feature vector was generated and transferred to the classification and regression layers. In the classification layer, the Softmax classifier was used to discriminate the foreground and background of the anchor points. In the regression layer, the center coordinates, the length, and the width of the anchor borders were adjusted to fit the candidate frame positions. The RPN multi-task loss function is:

(3) $$L\left(p_{i},t_{i}\right)=\frac{1}{N_{cls}}\sum_{i}L_{cls}\left(p_{i},p_{i}^{*}\right)+\lambda \frac{1}{N_{reg}}\sum_{i}p_{i}^{*}L_{reg}\left(t_{i},t_{i}^{*}\right)$$

where *i* is the anchor index; *p_i_* is the classification probability of anchor *i*; $p_{i}^{*}$ is the ground- truth for anchor *i*; *t_i_* is the difference between the prediction bounding box and the ground-truth label box in four parameter vectors (the horizontal, vertical coordinate value of the center point in the bounding box; the width and height of the bounding box); $t_{i}^{*}$ is the difference between the ground-truth label box and the positive anchor; *N_cls_* is the anchor size of mini-batch; *N_reg_* is the anchor position number; *L_cls_* is the log loss over two classes (object vs. not object); *L_reg_* is the regression loss, activated only if the anchor actually contains an object; *λ* is the balance parameter, 10 in this paper.

#### 2.4.5. Target Detection and Instance Segmentation

Target detection result output layer includes classification branch, regression branch, and FCN (Fully Convolutional Network). The classification branch calculates the classification loss of target and true label. The regression branch calculates the regression loss of the predicted frame and the true bounding box. The FCN branch was used for RoI pixel computation, where the RoI was up-sampled by the deconvolution layer to recover to the original image size. The average binary cross-entropy of the RoI region pixel values with the original image region pixel values was computed. The multi-task loss on each sampled RoI is:

(4) $$L=L_{cls}+L_{reg}+L_{mask}$$

(5) $$L_{mask}=-\sum_{i=1}^{N}y^{\left(i\right)}\mathrm{lg}{\hat{y}}^{\left(i\right)}+\left(1-y^{\left(i\right)}\right)\mathrm{lg}\left(1-y^{\left(i\right)}\right)$$

where *L* is model total loss function; *L_mask_* is the average binary cross-entropy loss; *N* is sample size; *y*^(*i*)^ is expected output for anchor *i*; ${\hat{y}}^{\left(i\right)}$ is actual output of anchor *i*.

Each ROI predicts an output of *K* * m^2^ dimensions through the mask branch and it encodes *K* binary masks with a resolution of m*m, corresponding to *K* classes. Then each pixel was classified by the activation Sigmoid function. The classification branch calculates the category of the candidate region and the regression branch calculates the location bounding box of the candidate region. The class, location, and profile of the impurity could finally be detected by the FCN.

#### 2.4.6. Precision and Recall

The precision rate (*P*), recall rate (*R*), average precision (*A_p_*), and comprehensive evaluation indicator *F*_1_ were applied to evaluate the impurity detection:

(6) $$P=\frac{T_{p}}{T_{p}+F_{p}}\times 100\%$$

(7) $$R=\frac{T_{p}}{T_{p}+F_{N}}\times 100\%$$

(8) $$A_{p}=\int_{0}^{1}P\left(R\right)dR$$

(9) $$F_{1}=2\frac{P\cdot R}{P+R}\times 100\%$$

where *T_P_* is the number of cases that are positive and detected positive; *F_P_* is the number of cases that are negative but detected positive; *F_N_* is the number of cases that are positive but detected negative; *P*(*R*) is the precision rate corresponding to each recall rate.

### 2.5. Impurity Rate Transformation Model

**Figure 7:**
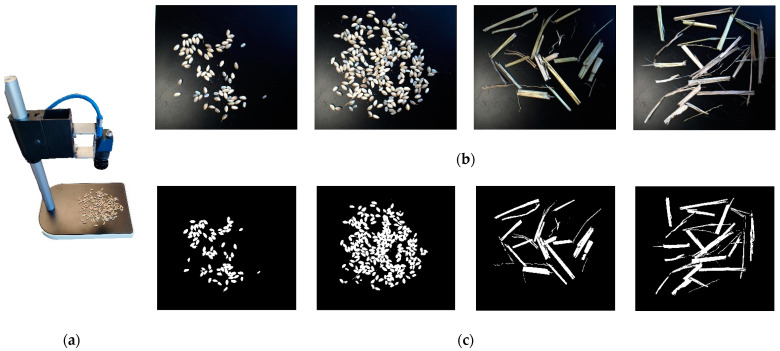
Material pixel density calibration test. (**a**) Calibration device; (**b**) calibration image; (**c**) binarized image.

As the material background (belt) is stable, crop pixels containing rice and impurity could be obtained through BackgroundSubtractorMOG (a function in opencv) by subtracting the background from the acquired image. The number of rice pixels is the number of grain pixels minus the number of impurity pixels. After obtaining the pixel number of each material (rice and impurity) in the grain, the material pixel density (mass per pixel) is needed to convert the pixel number into an actual mass to calculate the impurity rate. The pixel density of rice and impurity was acquired by calibration test as shown in Figure 7. A certain mass of rice and impurity (the mass of each component is shown in Figure 17) is separately weighed and spread without obscurity on the calibration device and the object distance of the camera was the same as the developed sampling device. The acquired images were binarized with a background grayscale of 0 and a material (rice, impurity) grayscale value of 255. The number of pixels with a grayscale value of 255 was counted and the relationship with the material quality was proportionally fitted. The slope of the fitted equation was the pixel density of the material. The impurity rate was calculated as:

(10) $$Z_{s}=k\frac{\rho_{i}\cdot p_{ni}}{\rho_{r}\cdot p_{nr}+\rho_{i}\cdot p_{ni}}\times 100\%$$

where *Z_s_* is impurity rate; *ρ_i_* is the pixel density of impurity, g/Pixel; *ρ_r_* is the pixel density of rice, g/Pixel; *p_ni_* is pixel number of impurity, Pixel; *p_nr_* is pixel number of rice, Pixel; *k* is correction factor of impurity rate.

To address the impact of material variation and moisture content on pixel density, correction factor of impurity rate *k* was proposed to compensate the pixel density differences due to material variability. In practical application, the correction factor of impurity rate *k* was acquired through the pre-test. Sample of rice and impurity was collected in advance in the field. The actual impurity rate *Z_s_*_1_ was obtained after manual sieving and weighing. After mixing, the material was put into the sampling device to obtain impurity rate *Z_s_*_2_. The correction factor of impurity rate was calculated as:

(11) $$k=Z_{s1}/Z_{s2}$$

### 2.6. Bench Test

**Figure 8:**
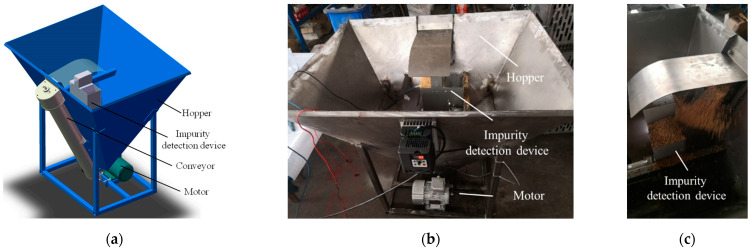
Bench test. (**a**) Test bench 3D model; (**b**) test bench physical picture; (**c**) material collection.

There are many varieties of rice in China and the differences among varieties in grain size and one thousand-grain mass are significant. To test the adaptability of the designed system to different varieties of rice, a bench test was carried out in the laboratory using a testing device shown in Figure 8, which could simulate the grain entering the grain bin from the conveyor. Thus, 5 rice varieties were used in the bench test, which were Lindao 20, Nanjing 40, Taijing 1105, Ningjing 5, and Liangyou 106. The abovementioned varieties were obtained in the harvest season. The impurity correction coefficients were calibrated by sampling before the tests. A certain mass of rice and impurity was weighed to obtain the actual impurity rate and then mixed smoothly into the hopper. The average detection impurity rate after a period of circulation was calculated and compared to the actual impurity rate. Each variety was repeated three times for the average and a total of 15 sets of tests was conducted.

### 2.7. Field Test

The field experiment was carried out in Hongqi Farm, Taizhou City (119.923° E, 32.456° N), Jiangsu Province, November 2021. The test machine was Thinker 4LZ-5.0 type full-feed tracked combine harvester (Huzhou, Zhejiang, China). The harvested rice variety is the “Ningjing 5”. Its basic properties were: average plant height of 1,003 mm, a yield of 9100.5 kg/hm^2^, grain moisture content 27.1%, one-thousand seed mass of 26.7 g. Before the test, the impurity correction coefficients were calibrated by sampling rice and impurity. The harvesting distance was 25 m and 5 groups of the test with different forward speed were carried out. The tracked combine harvester forward speed was 0.5 m/s~1.3 m/s in increments of 0.2 m/s (since the forward speed can only be controlled manually, the actual forward speed fluctuates slightly). Each speed was repeated 3 times for average and 15 sets of tests were conducted. After each test, no less than 2 kg of sample from different locations was taken from the combine harvester grain bin and weighing mass *m*_1_. The sample was cleaned and weighed again after the impurity was removed, *m*_2_. The impurity rate of manual detection can be calculated as:

(12) $$Z_{m}=\left(m_{1}-m_{2}\right)/m_{1}\times 100\%$$

where *Z_m_* is impurity rate of manual detection.

## 3. Result and Discussion

### 3.1. Effect of Light Irradiation

**Figure 9:**
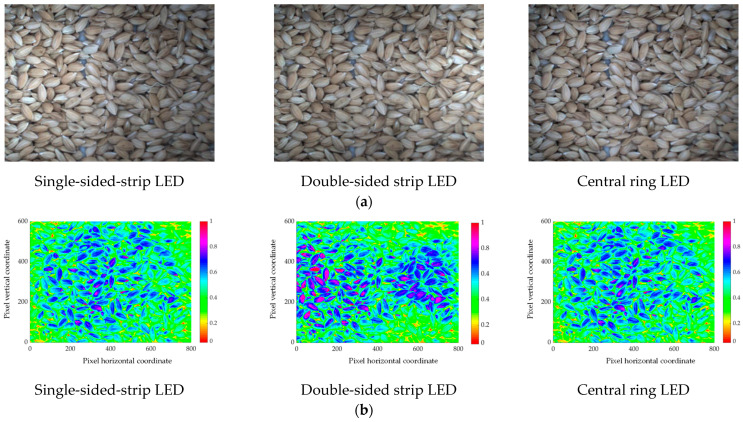
The grain images and the *V* component distribution under different light irradiations. (**a**) Grain images captured under different light irradiations; (**b**) the distribution of the *V* component.

The grain images captured under different light irradiations are shown in Figure 9a. The distributions of the *V* component are shown in Figure 9b. Under the single-sided-strip LED, the *V* value on the LED side was significantly higher than on the other and the distribution of the *V* value was disproportionate. Under the double-sided-strip LED, the *V* value was higher on the two sides and lower in the central region. Under the central-ring LED, the peripheral *V* value was lower than the central area.

**Figure 10:**
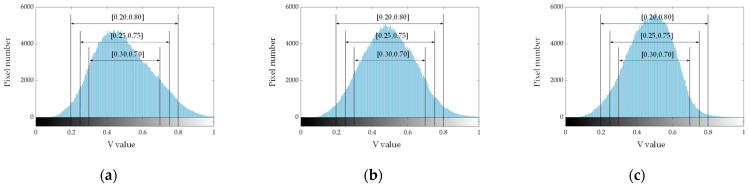
*V* value distribution histogram under light irradiation. (**a**) Single-sided-strip LED; (**b**) double-sided-strip LED; (**c**) central-ring LED.

To further quantify and analyze the effect of light irradiation on the image brightness, the *V* value of each pixel in the image was counted. The *V* value distribution histogram under light irradiation is shown in Figure 10. The higher the proportion of lightness around the median value (*V* = 0.5) and the smaller the coefficient of variation of the overall lightness, the more suitable it is for subsequent image processing. The *V* value percentage in a range of [0.30, 0.70], [0.25, 0.75], and [0.20, 0.80] and the coefficient of variation of the *V* value were calculated and the results are shown in Table 4.

According to Figure 10 and Table 4, under the single-sided-strip LED, the percentage of *V* value in a range of [0.30, 0.70], [0.25, 0.75], and [0.20, 0.80] was the smallest, which was 83.5%, 92.0%, and 96.6%, respectively. Under the central-ring LED, the percentage of *V* value in a range of [0.30, 0.70], [0.25, 0.75], and [0.20, 0.80] was the highest, which was 83.5%, 92.0%, and 96.6%, respectively. The coefficient of variation of the *V* value under different light irradiation was 0.311, 0.301, and 0.271, respectively. The *V* value coefficient of variation was minimized and the proportion of lightness around the median value was higher under the central-ring LED. Therefore, the image lightness distribution was most uniform under the central-ring LED, which was more conducive to the subsequent impurity recognition.

### 3.2. Effect of the Deflector Gap

#### 3.2.1. Effect on Impurity Visualization

**Figure 11:**
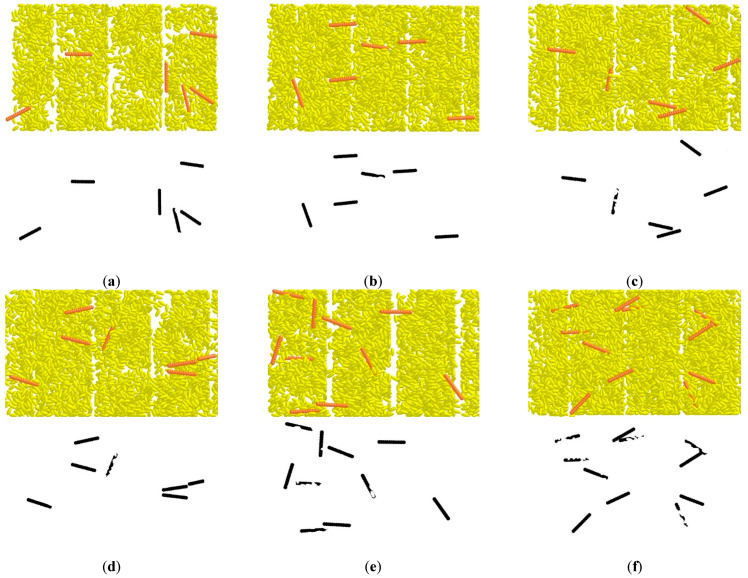
Impurity visualization analysis under different deflector gap. (**a**) *d* = 7.5 mm, *S* = 2.73%, (**b**) *d* = 10 mm, *S* = 3.75%, (**c**) *d* = 12.5 mm, *S* = 6.92%, (**d**) *d* = 15 mm, *S* = 9.54%, (**e**) *d* = 17.5 mm, *S* = 12.10%, (**f**) *d* = 20 mm, *S* = 18.11%.

The visualization of the impurity on the conveyor belt under different deflector gaps is shown in Figure 11. The average occlusion rate of impurity was 2.73% to 18.11% when the deflector gap varied from 7.5 mm to 20 mm. The occlusion rate increased with the increasing of the deflector gap. The occlusion of impurity mainly includes covered by rice and mutual occlusion between impurity. This is because when the deflector gap is small, the material could be transported in a thinner layer or even in a single layer and there is little intersection and overlap between impurity and rice. The impurity is partially or completely covered when the material layer is thick. The occlusion of impurity decreases with the decreasing grain thickness and cannot be identified through the vision system. Taking the occlusion rate of impurity less than 10% as the design standard, the deflector gap should meet the condition that *d* ≤ 15 mm.

#### 3.2.2. Effect on the Grain Distribution

**Figure 12:**
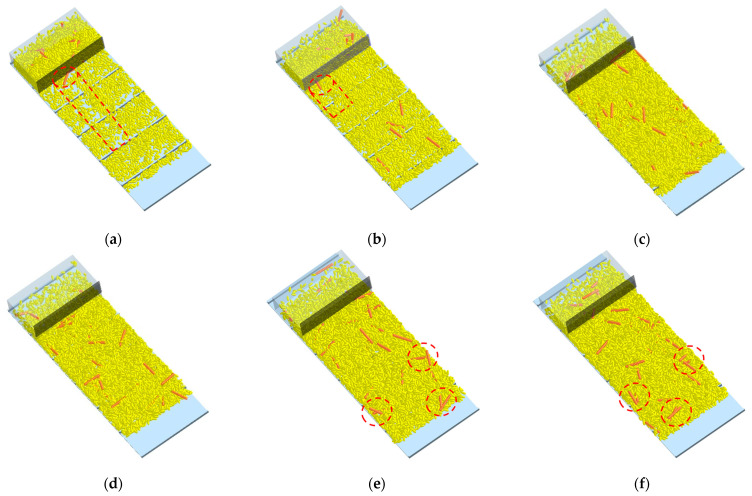
Grain distribution analysis under different deflector gap. (**a**) *d* = 7.5 mm; (**b**) *d* = 10 mm; (**c**) *d* = 12.5 mm; (**d**) *d* = 15 mm; (**e**) *d* = 17.5 mm; (**f**) *d* = 20 mm.

The grain distribution on the conveyor belt under different deflector gaps is shown in Figure 12. When deflector gap *d* ≤ 10 mm, the impurity was blocked and accumulated at the deflector. This accumulation also affects the passibility of the grain, leaving gaps in the conveyor belt (Figure 12a,b). However, intersection and overlap between impurity would occur when *d* ≥ 17.5 mm (Figure 12e,f), which indicates that two layers of impurity could be passed under the gap. This is consistent with the result in Figure 11. To ensure the grain passability and without impurity multi-layer accumulation at the same time, the deflector gap should meet the condition that 12.5 mm ≤ *d* ≤ 15 mm.

#### 3.2.3. Effect on the Grain Mass Flow Rate

**Figure 13:**
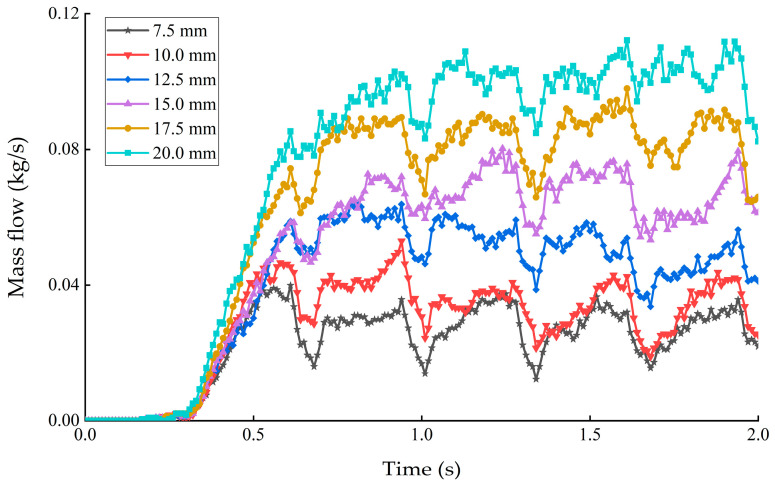
Grain mass flow rate under different deflector gaps.

The grain mass flow rate under different deflector gaps is shown in Figure 13. The average mass flow rate of grain was 0.04 kg/s to 0.15 kg/s when the deflector gap varied from 7.5 mm to 20 mm. The grain mass flow rate increases with the increasing of deflector gap. The grain mass flow rate decreases as the corrugation passes through the deflector. The grain mass flow rate was uniform and stable in general, which is beneficial for impurity identification.

#### 3.2.4. Simulation Validation

**Figure 14:**
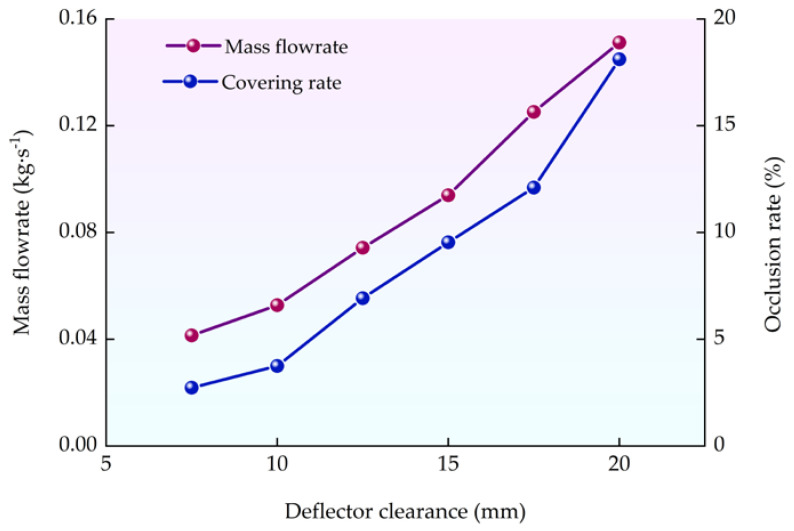
The variation curve of impurity occlusion rate and grain mass flow rate with the deflector gap.

Combining the above results, the variation in impurity occlusion rate and grain mass flow rate with the deflector gap is shown in Figure 14. The grain mass flow rate and impurity occlusion rate both increase when the deflector gap increases. The detection efficiency could be improved by enhancing the deflector gap but the impurity identification accuracy decreases at the same time. The deflector gap was determined to be 15 mm to ensure detection accuracy while maximizing sampling efficiency. The sampling efficiency was 0.094 kg/s at this deflector gap.

To verify the simulation model, the mass flow rate comparison test was carried out. After the grain flow was uniform, the sample box was placed at the sampling device outlet. The test result showed that a total of 0.87 kg of grain was collected in 10 s and the actual grain mass flow rate was 0.087 kg/s. The simulation flow rate of 0.094 kg has an error of 7.2% with the physical test, which demonstrates that the simulation model is reliable.

### 3.3. Impurity Segmentation

**Figure 15:**
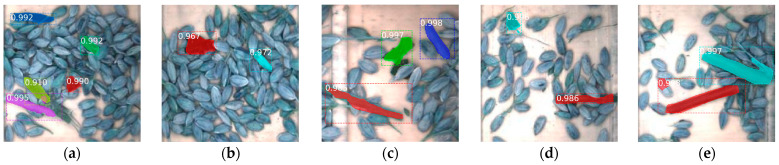
Impurity segmentation result. Masks are shown in color and confidences are also shown. (**a**) High impurity rate and dense rice; (**b**) low impurity rate and dense rice; (**c**) high impurity rate and sparse rice; (**d**) low impurity rate and sparse rice; (**e**) large size impurity.

The detection model was trained in the Anaconda3 virtual environment. The Keras 2.1.6 and TensorFlow 1.15.0 were used to build the deep learning framework in the Python 3.7 environment. The training process used a migration learning method to initialize the network using Mask R-CNN COCO pre-trained parameters. Each batch size was processed with two images. The grain images of the test set were used to validate the identification model. The number of training steps per epoch, the learning rate, and the skip detection confidence were set to 100, 0.001, and 90%, respectively. The training time of the 1200 grain images was 84 h and the average identification time of a single image was 1.58 s. The impurity detection results under different mass flow rates are shown in Figure 15.

It can be seen from Figure 15 that the impurity could be identified accurately by the system in general. In case of an overlap between rice and impurity, they can be distinguished following the impurity contour boundary. The precision rate (*P*), recall rate (*R*), average precision (*A_p_*), and comprehensive evaluation indicator *F*_1_ were 92.49%, 88.63%, 81.47%, and 90.52%. It is difficult to identify when the impurity was relatively fine and small.

**Figure 16:**
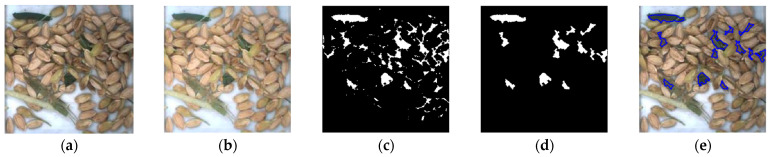
Impurity identification based on color space and morphology. (**a**) Original image; (**b**) image equalization; (**c**) threshold segmentation; (**d**) removing interference; (**e**) identification result.

For the purpose of contrasting with the traditional method of identifying rice by color space and morphology [30], comparative tests were conducted. The grain image in Figure 16a was converted to HSV color space, and the histogram equalization of the V-component of the image was obtained in Figure 16b. The range of color threshold distribution of impurity components was extracted manually, and the morphological closure operation was used to obtain Figure 16c after color extraction of the image. Via shape feature analysis, the targets with a large ratio of perimeter to the area were deleted and the processing result is shown in Figure 16d. Finally, the detection results are marked on the original image, as shown in Figure 16e.

According to Figure 16, the impurity that was close to the rice color failed to be detected. The gaps between the rice are close to the color and morphology of the impurity and are easily misidentified as an impurity. The impurity-detection method proposed based on Mask R-CNN has better adaptability when the color features are close to the background and the distribution area adheres to the background.

### 3.4. Pixel Density Calibration

**Figure 17:**
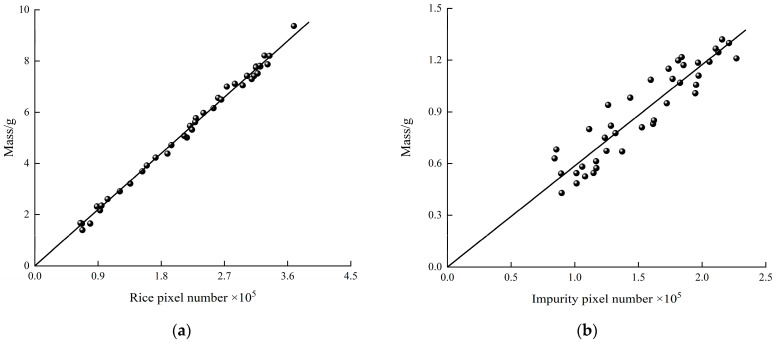
The relationship between pixel number and mass. (**a**) Rice; (**b**) impurity.

The least squares was used to linearly fit the relationship between pixel number and mass of rice and impurity. The intercept was set to 0 to avoid lack of fit when the impurity mass was small. The relationships between pixel number and mass of rice and impurity are shown in Figure 17.

The regression equations are:

*y*_1_ = 2.4401 × 10^−5^
*x*_1_ (13)

*y*_2_ = 0.5867 × 10^−5^
*x*_2_ (14)

where *x*_1_ is pixel number of rice; *x*_2_ is pixel number of impurity, g; *y*_1_ is mass of rice, g; *y*_2_ is mass of impurity, g.

R-square (coefficient of determination) and RMSE (root mean squared error) were used to evaluate the fitting results. The R-square and RMSE of regression Equation (13) are 0.9949 and 0.1626, and regression Equation (14) is 0.8604 and 0.1013, respectively. Therefore, the rice pixel density *ρ_r_* and impurity pixel density *ρ_i_* are 2.4401 × 10^−5^ g/Pixel, 0.5867 × 10^−5^ g/Pixel, respectively. The accuracy for the rice pixel density is higher than that of impurities. It is because the proportion of different types of impurities affects the pixel density.

### 3.5. Bench Test

The bench test result is shown in Table 5, from which the designed system has a mean detection accuracy of 91.15~97.26% for the five varieties and, therefore, has good applicability to the rice of different varieties. The correction factor of impurity rate *k* varied from 0.912 to 1.075 due to the rice variety and moisture. After compensation by the correction factor of impurity rate *k*, the system could successfully calculate the actual impurity for different rice varieties. In a stable indoor environment, the average recognition accuracy of the system was 94.47%. The source of error may be the material’s irregular three-dimensional morphology. The calculated mass using surface density varies from the actual mass. During manual detection, despite the multi-point sampling method being used, the samples collected manually cannot be identical to those taken by the sampling device. This inevitably leads to errors in manual detection and system detection.

### 3.6. Field Test

The field test result is shown in Table 6. It can be seen that the results’ relative error was in a range of 5.71~11.72% and average relative error was 8.39% between the impurity-detection system and the manual method. Compared with the bench test, the average identification accuracy in the field test was reduced by 2.86%. The main reason may be the variability in the material state in the field. Despite the impurity correction coefficient *k* being used to compensate for the material variation and moisture content on pixel density, there were still differences in materials between different areas in the field. The working vibration of the harvester also affect the test results. In the field environment, the system recognition accuracy had little to do with the forward speed. Generally, the impurity identification accuracy in the field was not far from the bench test, which indicates that the designed infusion-type sampling device and impurity recognition algorithm are able to adapt to the field conditions.

## 4. Conclusions

In this paper, an infusion-type sampling device and impurity recognition algorithm based on Mask R-CNN were developed to monitor tracked rice combine harvester impurity rate in real time. Obscured contaminants can be reduced by the infusion-type sampling device. Impurity in grains could be efficiently identified and converted to the actual impurity rate by pixel density. The specific work is summarized as:

(1) To reduce the obstruction of impurity, an infusion-type sampling device was developed. The image lightness distribution under different light irradiations was investigated. The results show that the image under the central-ring LED had the smallest most uniform brightness distribution and is the superior light source. The variation coefficient of brightness was 0.271. According to the DEM simulation of the grain transportation process, the effect of the deflector gap on impurity visualization, grain passibility, and mass flow rate was analyzed. The deflector gap is determined to be 12.5~15.0 mm, which reduces the impurity obscuration and ensures the passibility of the grain.
(2) To overcome the misidentification caused by color and morphology proximity, the impurity recognition algorithm based on Mask R-CNN was proposed. The test set experiment showed that the precision rate, recall rate, average precision, and comprehensive evaluation indicator were 92.49%, 88.63%, 81.47%, and 90.52%. The pixel densities of rice and impurities were obtained by calibration tests and least-squares fitting. The fitting equation R-square for rice and impurity was 0.9949 and 0.8604, respectively. The correction factor of impurity rate was used to correct pixel density variation caused by variety and moisture content.
(3) The bench test results show that the designed system has a good detection accuracy of 91.15~97.26% for the five varieties. The results’ relative error was in a range of 5.71~11.72% between the impurity-detection system and manual method in field conditions.

The accuracy of impurity rate detection is related to the training sample size. In future research, the number of learning samples will be increased and the model structure will be further optimized to improve the ability of impurity recognition. A lightweight neural network will be used for feature extraction to improve the real-time performance of impurity detection and reduce calculation capacity to utilize the embedded systems.

## Figures and Tables

**Table 1:** Properties of materials used in EDEM.

Material	Density (kg/m^3^)	Poisson’s Ratio	Shear Modulus (MPa)
Rice	1350	0.3	180
Impurity	198	0.4	48
Belt	2500	0.49	2
Shell	7800	0.33	80,000

**Table 2:** Contact parameters used in the EDEM for grain contact model simulation.

Types	Collision Recovery Coefficient	Static Friction Coefficient	Dynamic Friction Coefficient
Rice-rice	0.19	0.81	0.05
Rice-impurity	0.17	0.80	0.03
Rice-belt	0.42	0.50	0.01
Rice-shell	0.52	0.45	0.01
Impurity-belt	0.09	0.60	0.02
Impurity-shell	0.10	0.66	0.02
Impurity-impurity	0.23	0.44	0.07

**Table 3:** ResNet-101 network architecture.

Layer Name	Output Size	Convolution Kernel
Conv1	112 × 112	7 × 7, 64, stride 2 3 × 3 max pool, stride 2
Conv2_x	56 × 56	$\left[\begin{array}{cc} 1\times 1 & 64\\ 3\times 3 & 64\\ 1\times 1 & 256 \end{array}\right]$ × 3, stride 2
Conv3_x	28 × 28	$\left[\begin{array}{cc} 1\times 1 & 128\\ 3\times 3 & 128\\ 1\times 1 & 256 \end{array}\right]$ × 4, stride 2
Conv4_x	14 × 14	$\left[\begin{array}{cc} 1\times 1 & 256\\ 3\times 3 & 256\\ 1\times 1 & 256 \end{array}\right]$ × 23, stride 2
Conv5_x	7 × 7	$\left[\begin{array}{cc} 1\times 1 & 512\\ 3\times 3 & 512\\ 1\times 1 & 2048 \end{array}\right]$ × 3, stride 2

**Table 4:** *V* value distribution indicator.

*V* Value Indicator	Light Irradiation		
	Single-Sided-Strip LED	Double-Sided Strip LED	Central Ring LED
Percentage in the range of [0.30, 0.70]	83.5%	86.6%	91.2%
Percentage in the range of [0.25, 0.75]	92.0%	93.8%	96.2%
Percentage in the range of [0.20, 0.80]	96.6%	97.5%	98.6%
Coefficient of variation	0.311	0.301	0.271

**Table 5:** Bench test result.

Varieties	Rice Mass (kg)	Moisture (%)	Impurity Mass (kg)	Correction Factor of Impurity Rate *k*	Actual Impurity Rate (%)	Detection Impurity Rate (%)	Detection Accuracy (%)
Lindao 20	11.55	22.7	0.32	0.968	2.8	2.64	94.33
Nanjing 40	9.83	28.7	0.41	0.912	4.2	4.34	96.72
Taijing 1105	12.21	25.5	0.41	0.936	3.3	3.53	92.91
Ningjing 5	10.98	26.9	0.31	0.950	2.8	2.88	97.26
Liangyou 106	11.58	24.8	0.42	1.075	3.6	3.28	91.15

**Table 6:** Field test result.

Test No.	Forward Speed (m/s)	Grain Mass *m*_1_ (kg)	Grain Mass without Impurity *m*_2_ (kg)	Impurity Rate of Manual Detection (%)	Impurity Rate of System Detection (%)	Detection Error (%)
1	0.53	3.079	3.01	2.25	2.40	8.13
2	0.71	3.293	3.22	2.44	2.24	−9.46
3	0.89	2.606	2.55	1.96	2.08	6.95
4	1.15	3.279	3.23	1.57	1.50	−5.71
5	1.33	2.797	2.74	2.11	1.88	−11.72

## Data Availability

The data presented in this study are available on request from the authors.
